# Genome-Wide Identification of Associations of Circulating Molecules With Spontaneous Coronary Artery Dissection and Aortic Aneurysm and Dissection

**DOI:** 10.3389/fcvm.2022.874912

**Published:** 2022-04-27

**Authors:** Tianci Chai, Mengyue Tian, Xiaojie Yang, Zhihuang Qiu, Xinjian Lin, Liangwan Chen

**Affiliations:** ^1^Department of Cardiac Surgery, Fujian Medical University Union Hospital, Fuzhou, China; ^2^Fujian Key Laboratory of Cardio-Thoracic Surgery (Fujian Medical University), Fuzhou, China; ^3^Department of Anesthesiology, Xinyi People’s Hospital, Xuzhou, China; ^4^Key Laboratory of Ministry of Education for Gastrointestinal Cancer, School of Basic Medical Sciences, Fujian Medical University, Fuzhou, China; ^5^Department of Thoracic Surgery, Fujian Medical University Union Hospital, Fuzhou, China

**Keywords:** spontaneous coronary artery dissection, aortic aneurysm and dissection, circulating proteins, genome-wide association study, lipids, ECM1

## Abstract

Circulating proteins play functional roles in various biological processes and disease pathogenesis. The aim of this study was to highlight circulating proteins associated with aortic aneurysm and dissection (AAD) and spontaneous coronary artery dissection (SCAD). We examined the associations of circulating molecule levels with SCAD by integrating data from a genome-wide association study (GWAS) of CanSCAD and 7 pQTL studies. Mendelian randomization (MR) analysis was applied to examine the associations between circulating molecule levels and AAD by using data from UK Biobank GWAS and pQTL studies. The SCAD-associated SNPs in 1q21.2 were strongly associated with circulating levels of extracellular matrix protein 1 (ECM1) and 25 other proteins (encoded by *CTSS*, *CAT*, *CNDP1*, *KNG1*, *SLAMF7*, *TIE1*, *CXCL1*, *MBL2*, *ESD*, *CXCL16*, *CCL14*, *KCNE5, CST7*, *PSME1*, *GPC3*, *MAP2K4*, *SPOCK3*, *LRPPRC*, *CLEC4M*, *NOG*, *C1QTNF9*, *CX3CL1*, *SCP2D1*, *SERPINF2*, and *FN1*). These proteins were enriched in biological processes such as regulation of peptidase activity and regulation of cellular protein metabolic processes. Proteins (FGF6, FGF9, HGF, BCL2L1, and VEGFA) involved in the Ras signaling pathway were identified to be related to AAD. In addition, SCAD- and AAD-associated SNPs were associated with cytokine and lipid levels. MR analysis showed that circulating ECM1, SPOCK3 and IL1b levels were associated with AAD. Circulating levels of low-density lipoprotein cholesterol and small very-low-density lipoprotein particles were strongly associated with AAD. The present study found associations between circulating proteins and lipids and SCAD and AAD. Circulating ECM1 and low-density lipoprotein cholesterol may play a role in the pathology of SCAD and AAD.

## Introduction

Aortic aneurysm and dissection (AAD), characterized by intimal rupture and intramural hematoma formation, is a life-threatening disease that affects the aorta. AAD is associated with a high risk of mortality (1). With the growing use of imaging examinations, the detection of AAD has increased. The latest data from the Global Burden Disease 2010 project showed that the overall global death rate of AAD increased from 2.49 per 100,000 persons per year to 2.78 per 100,000 persons per year between 1990 and 2010 (2, 3). The risk of AAD increases with age, and men are more often affected than women (2). Until now, the occurrence and development of AAD have been unpredictable.

The identification of AAD with familial association has led to our knowledge of its genetically mediated physiopathological mechanisms. AAD can occur in association with a genetic syndrome or as an autosomal dominant disorder without syndromic features (4, 5). Familial aggregation of AAD has been demonstrated in previous genetic studies. Genetic loci for the condition have also been mapped (6–9). Pathogenic variants were found in *COL3A1* (10), *SMAD3* (10, 11), *TGFBR1/2*, *TGFB2/3* (12), *FBN1*, *PHACTR1/EDN1* (13), *TSR1* (14, 15), *TLN1* (16), and others (9, 16, 17).

Spontaneous coronary artery dissection (SCAD) is a rare event with an incidence of approximately 0.28–1.1% and occurs most commonly in women (18). In SCAD associated with atherosclerosis, inflammation and rupture of atherosclerotic plaque cause disruption of the intimal-medial junction of the artery and result in a false lumen in the arterial wall. Blood flow within the true lumen is then obstructed or restricted due to the squeeze of intramural hemorrhage and hematoma within the false lumen on the true coronary lumen (19). SCAD leads to myocardial infarction or sudden cardiac death. Although the risk factors for SCAD are not fully understood, hypertension, coronary atherosclerosis, coronary spasm related to cocaine use, peripartum state, Prinzmetal angina, and connective tissue disorders have been associated with dissection (20). A genome-wide association study (GWAS) discovered associations at chromosomes 1q21.2, 6p24.1, and 12q13.3 and associations for non-coding variants at chromosomes 12q13.3 and 21q22.11 for SCAD (17). Previous findings supported the complex genetic physiopathologic mechanisms of SCAD. However, although some of the genetic variants are known to affect gene expression, their regulatory pathways in SCAD and AAD and the risk factors are largely unknown.

Circulating proteins play functional roles in various biological processes and disease pathogenesis and have been suggested to be druggable targets (21, 22). Recently, published GWASs of circulating proteins have identified a large number of genetic variants associated with circulating proteins (protein quantitative trait loci, or pQTLs) (23–27). This genetic evidence offers the opportunity to systematically evaluate the causal effects of various potential drug targets on diseases. Indeed, the GWAS identified loci containing genes that encode proteins involved in arterial vascular wall structure and function. Circulating proteins such as TGF β receptor, collagen α1 chain, fibrillin, endotelin-1, ribosome maturation factor, and talin 1 were implicated in the development of SCAD (28). Cytokines such as the tumor necrosis factor superfamily, growth factor, interferon, interleukins, colony stimulating factor, and chemotactic factor play important roles in AAD.

**FIGURE 1:**
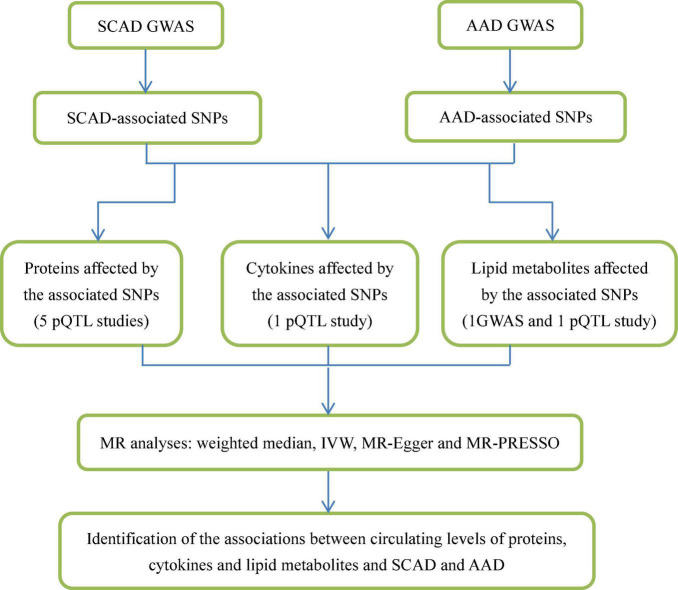
Flowchart of the study design. We designed this study to explore potential risk factors, such as circulating molecules, for SCAD and AAD. This study comprised several steps of analyses. In the first step, we identified SCAD- and AAD-associated SNPs from GWASs. Second, we looked for circulating proteins, cytokines and lipid metabolites associated with these SNPs using data from pQTL studies. Third, for circulating molecule levels that were significantly affected by SCAD- and AAD-associated SNPs, we applied 4 MR methods (weighted median, IVW, MR-Egger, and MR-PRESSO) to examine whether they were genetically associated with SCAD and AAD.

Circulating lipid composition plays a key role in AAD (29). The association between lipid levels and AAD risk has been examined in case–control study (30), but causal relationships have not been determined. Identification of regulatory variants in the susceptibility genetic loci and determination of the association between circulating molecules affected by these genetic variants and AAD and SCAD will further elucidate the physiopathologic mechanism. This study represents an effort to identify potential circulating molecules implicated in AAD and SCAD development by performing Mendelian randomization (MR) analysis (Figure 1).

## Materials and Methods

### Association Data for Spontaneous Coronary Artery Dissection and Aneurysm and Dissection

In this study, we determined genetic loci for SCAD according to the GWAS from the Canadian SCAD (CanSCAD) study (17), which contained 270 SCAD patients and 5,263 non-SCAD controls in the discovery stage of this study. The CanSCAD study samples were analyzed with control subjects derived from the Michigan Genomics Initiative biorepository with electronic health record-based phenotyping to exclude individuals with vascular diseases or connective tissue disorders. The summary results released from the GWAS included association *P*-values of 607,778 genotyped variants with SCAD (17), which can be downloaded from the NHGRI-EBI GWAS Catalog (accession number GCST90000582). The dataset contains six columns, which present the information of rs ID, chromosome, position, effect allele, non-effect allele and *P*-value for each SNP. SNPs with *P* < 1.0 × 10^–5^ were identified, and the SCAD loci were determined. The genome-wide significance threshold was set to 5.0 × 10^–8^ in this study.

GWAS summary data of AAD (ICD-10 code: I71), which contains 452,264 individuals from the UK Biobank, were obtained. GWASs for 778 traits in the UK Biobank were performed (31). The GWAS for AAD included 1,470 AAD patients and 450,794 controls. The analysis comprised 62,394 genotyped variants and 9,113,133 imputed variants that passed quality control. The linear mixed model was applied in the genome-wide association analysis to avoid bias caused by the related individuals. Detailed information on the study design, samples, genotyping method, genotype imputation, quality control, and genetic association analysis has been detailed in a published paper (32). A searchable atlas of genetic associations for the 778 traits in UK Biobank was made.^1^ The summary data for AAD can be found by searching “Aortic aneurysm and dissection” at this website. The downloaded dataset contains information on SNP ID, effect allele, non-effect allele, allele frequency, beta, standard error, and *p*-value for each SNP, which are necessary for the MR analysis. SNPs with minor allele frequencies greater than 0.05 in this dataset were used. These summary data were used in the MR analysis in this study.

### Association Data for Circulating Proteins

We carried out pQTL analysis in peripheral blood for the identified SCAD- and AAD-associated SNPs to identify their functional potentials. pQTL signals with *P*-values less than 1.0 × 10^–5^ were considered in this study. The pQTL analyses were performed in the five pQTL studies listed below. The downloaded datasets contain information on SNP ID, effect allele, non-effect allele, allele frequency, beta, standard error and *p*-value for each SNP, which are necessary for the MR analysis. Based on these pQTL data, circulating proteins associated with the SCAD- and AAD-associated SNPs were identified, and MR analyses were performed.

(i) The KORA study. This study quantified levels of 1,124 proteins in blood plasma samples of 1,000 individuals. Associations between genome-wide SNPs and the protein levels were examined; that is, 1,124 GWASs were performed (24). The genome-wide summary datasets are available at http://metabolomics.helmholtz-muenchen.de/pgwas/index.php?task=download. The data contain associations between 509,946 SNPs and 1,124 proteins.

(ii) The INTERVAL pQTL study.^2^ The INTERVAL study is a genomic bioresource of 50,000 blood donors from 25 centers across England. The pQTL study tested associations of 10.6 million imputed autosomal variants with levels of 2,994 circulating proteins in 3,301 healthy participants from the INTERVAL study (26).

(iii) The cardiovascular disease-relevant protein GWAS.^3^ This pQTL study is a GWAS that analyzed genome-wide associations between SNPs and 83 proteins considered relevant to cardiovascular disease measured in 3,394 individuals (23).

(iv) The Framingham Heart Study. This pQTL study published 16,602 pQTL results for 71 high-value cardiovascular disease proteins that were measured in 7,333 Framingham Heart Study participants (27).

(v) The AGES Reykjavik study. A total of 1,046 significant pQTL results were obtained from this pQTL study. This study measured 4,137 proteins on the Slow-Off rate Modified Aptamer (SOMAmer)-based proteomic profiling platform (SOMAscan) in 5,457 Icelanders (25). Associations of 27.2 million sequence variants with the levels of 4,907 plasma proteins were examined, and the summary data are publicly available.^4^

### Association Data for Cytokines

Studies of the link between cytokines and growth factors, which are regulators of inflammation, and AAD could deepen our understanding of the disease and may guide future treatment therapies. Therefore, we searched for links between AAD-SNPs and cytokine levels. A pQTL study examined the genome-wide associations between 10.7 million SNPs and 41 cytokine concentrations in up to 8,293 Finnish individuals (33). Based on this pQTL study, cytokines associated with the SCAD- and AAD-associated SNPs were identified.

### Association Data for Circulating Metabolites

Blood metabolites could potentially serve as biomarkers for SCAD and AAD. Comprehensive molecular profiling of circulating metabolites can help to uncover the metabolic pathophysiology underlying SCAD- and AAD-associated variants. We identified associations between SCAD- and AAD-SNPs and circulating metabolites. A total of 123 metabolites that represent a broad molecular signature of systemic metabolism in blood were quantified using the quantitative high-throughput nuclear magnetic resonance (NMR) metabolomics platform in up to 24,925 individuals. Associations between 39 million genetic markers and the metabolite concentrations were examined and the summary data are publicly available (34).

### Functional Enrichment Analysis

We then carried out gene ontology (GO) analysis. GO contains three independent ontologies: biological process, molecular function and cellular component (35). GO analyses were used to explore the biological properties of the genes coding the identified proteins. The Database for Annotation, Visualization and Integrated Discovery (DAVID) bioinformatic tool^5^ was used to carry out the GO analysis and illustrate the functional enrichments (36).

### Mendelian Randomization Analysis

For the identified proteins, we performed MR analyses based on the GWAS summary data to test if circulating levels of the molecules identified in the pQTL studies were associated with AAD. The association between circulating protein levels and SCAD was not examined because some necessary data (e.g., beta and standard error) are unavailable. The weighted median (37), inverse-variance weighted (IVW) (38), MR–Egger (39), and MR pleiotropy residual sum and outlier (MR-PRESSO) (40) methods were applied and the results of IVW and MR–Egger analysis were mainly considered. The weighted median estimator gives a credible estimate of effect when up to half of the weight is derived from valid instrumental variables. The IVW method combines the ratio estimates from each instrumental variable in a meta-analysis model (38). If horizontal pleiotropy exists, the intercept from MR–Egger analysis would be expected to differ from zero (39). On this occasion, the causal estimates from MR–Egger analysis were chosen. Conversely, in the absence of horizontal pleiotropy, the causal estimates from IVW MR analyses were used, as they retain greater power. We also detected horizontal pleiotropy and outlier-corrected causal estimation by using MR-PRESSO tests (40). The outlier test in MR-PRESSO is the procedure to test for the MR assumption of no pleiotropy.

The data required in the MR analysis (i.e., the SNP rs number, beta values, standard errors, and *P*-values) were extracted from the summary datasets of GWAS and pQTL studies described above. In the pQTL summary data, SNPs with a *P*-value less than 1.0 × 10^–5^ were selected as potential instrumental variables. The selection criterion was not set to 5.0 × 10^–8^ because this stringent threshold would lead to too few instrumental variables, which may hinder the analyses. We clumped SNPs (linkage disequilibrium *r*^2^ < 0.01 within 10,000 kb) based on data from Europeans from the 1,000 Genomes project using the “clump_data” function in the R package TwoSampleMR to select independent instrumental variables. The consistency of the effect allele for each SNP in the BP GWAS and pQTL studies was checked and the direction of the effect was corrected when inconsistency existed. The IVW MR and MR–Egger analyses were performed by using the MendelianRandomization R package (41). Multivariable MR analyses were performed by applying the multivariable IVW method. The source code and documents for MR-PRESSO are available at https://github.com/rondolab/MR-PRESSO. The default parameters were used for the MR-PRESSO analysis.

### The Causal Relationships Between Lipid Concentrations and Aneurysm and Dissection

We also evaluated the effects of lipid concentrations on AAD. Summary statistics from the Global Lipids Genetics Consortium (GLGC) GWAS, which identified more than one hundred lipid-associated loci, were applied in the present study (42, 43). This GWAS examined the associations between 2.6 million SNPs and total cholesterol (TC), triglyceride (TG), high-density lipoprotein cholesterol (HDL-c), and low-density lipoprotein cholesterol (LDL-c) levels in 188,578 individuals of European descent. We applied summary data from the GLGC GWAS and UK Biobank AAD GWAS in MR analysis to evaluate the causal associations between blood lipid concentrations and AAD. To further support the relationship between blood lipid concentrations and AAD, GWAS summary data for “high cholesterol” (ICD-10 code: 1536) and “Disorders of lipoprotein metabolism and other lipidaemias” (ICD-10 code: E78) from the UK Biobank (see text footnote) were also obtained, and the associations between high cholesterol and lipidemia and AAD were examined in MR analyses. Information on GWASs for 778 traits in UK Biobank has been described above (31). The “high cholesterol” GWAS included 55,265 cases and 396,999 controls. The “Disorders of lipoprotein metabolism and other lipidaemias” GWAS included 39,308 cases and 412,956 controls.

### Identification of Potential Functional Variants

The identified pQTLs may link proteins to SCAD and AAD risk. However, these variants may be associated with gene expression levels and protein levels largely due to linkage. Therefore, we still need to distinguish functional variants from others. We searched for missense mutations, N^6^-methyladenosine (m^6^A)-associated SNPs^6^ (44), phosphorylation-related SNPs^7^ (phosSNPs) (45) and SNPs that alter the activity of putative regulatory elements to find potential functional variants. SNPs with altered enhancer and promoter activity were also identified in the survey of regulatory elements (SuRE) data browser database^8^ (46).

### Statistical Analysis

The data applied in this study were extracted from publicly available summary statistics files. The weighted median, IVW and MR–Egger tests were carried out using the R package “MendelianRandomization” (41). Statistical power was estimated by applying the method proposed by Stephen Burgess (47). We tested the associations between approximately 200 molecules and AAD in MR analysis, so we set the significance threshold to 2.50 × 10^–4^. A false positive rate less than 0.05 was determined to be significant in functional enrichment analysis. All the figures were drawn by using the R language.

## Results

### The Spontaneous Coronary Artery Dissection—and Aneurysm and Dissection-Associated Loci

According to the SCAD GWAS summary data, we picked up 13 SCAD-associated loci, including 1p32.1, 1q21.2, 2p23.2, 2q33.2, 4q34.3, 5q23.2, 6p24.1, 6q22.1, 6q25.3, 12q13.3, 12q21.33, 12q23.2, and 21q22.11 (Table 1). The top signals were located in 1q21.2, in which 149 SNPs showed significant associations with SCAD (*P* < 5.0 10^–8^). The top SNP associated with SCAD in 1q21.2 was rs12740679 (*P* = 2.19 × 10^–12^). There were fewer genome-wide significant SNPs in other selected loci. Four SNPs, rs11172113 (*P* = 2.63 × 10^–8^), rs10747776 (*P* = 4.92 × 10^–8^), rs4367982 (*P* = 2.77 × 10^–8^), and rs4759277 (*P* = 4.77 × 10^–8^), in 12q13.3 and one SNP (rs9349379, *P* = 4.36 × 10^–8^) in 6p24.1 were significantly associated with SCAD. For the remaining ten selected loci, no SNP reached the genome-wide significance level. For 2p23.2, 4q34.3, 5q23.2, and 12q21.33, only one SNP reached 1.0 × 10^–5^ in each locus. In 6q25.3, 11 SNPs passed 1.0 × 10^–5^. The top SNP in 6q25.3 was rs78349783 (1.03 × 10^–6^). SNPs in these 13 loci were considered in the following analysis.

**TABLE 1:** The genomic loci collected from GWAS for SCAD and AAD.

Disease	Loci	Number of SNPs with *P*-value < 1.0 × 10^–5^	Number of SNPs with *P*-value < 5.0 × 10^–8^	Top SNP	
				rs ID	*P*-value
SCAD	1p32.1	7	0	rs11207415	3.23E-06
SCAD	1q21.2	291	149	rs12740679	2.19E-12
SCAD	2p23.2	1	0	rs4535004	8.32E-06
SCAD	2q33.2	3	0	rs78377252	2.43E-06
SCAD	4q34.3	1	0	rs2715408	7.64E-06
SCAD	5q23.2	1	0	rs17839701	9.94E-06
SCAD	6p24.1	5	1	rs9349379	4.36E-08
SCAD	6q22.1	3	0	rs7775726	6.94E-06
SCAD	6q25.3	11	0	rs78349783	1.03E-06
SCAD	12q13.3	14	5	rs11172113	2.63E-08
SCAD	12q21.33	1	0	rs2722224	6.71E-06
SCAD	12q23.2	3	0	rs1014675	5.45E-06
SCAD	21q22.11	3	0	rs28451064	1.19E-07
AAD	1q41	2	0	rs1417488	5.05E-06
AAD	2q22.1-q22.2	1	0	rs1523704	8.89E-06
AAD	3p26.1	3	0	rs4686009	7.72E-06
AAD	3q26.33	3	0	rs34234720	7.38E-07
AAD	5q14.1	1	0	rs10042584	2.89E-06
AAD	6q16.1	10	0	rs11968306	5.63E-06
AAD	6q16.3	3	0	rs12213917	7.64E-06
AAD	6q27	1	0	rs221727	3.85E-06
AAD	7p15.3	1	0	rs2188989	5.59E-06
AAD	7p14.1	1	0	rs7804009	8.12E-06
AAD	7p11.2	50	0	rs2634083	1.50E-06
AAD	7q36.2	3	0	rs73165230	3.64E-06
AAD	8p11.22	1	0	rs4595091	3.52E-06
AAD	9q22.31	1	0	rs10991774	8.58E-06
AAD	10q21.3	1	0	rs189204293	3.97E-06
AAD	11p15.4	50	4	rs112809417	4.10E-08
AAD	11p14.2	1	0	rs2703420	9.13E-06
AAD	11q24.1	15	0	rs17338045	1.44E-07
AAD	11q24.3	11	0	rs7936928	3.16E-06
AAD	13q12.11	10	0	rs9506822	1.87E-06
AAD	13q34	1	0	rs2039092	8.49E-06
AAD	14q12	8	0	rs3825741	5.01E-06
AAD	14q24.3	7	0	rs3784004	3.36E-06
AAD	15q25.1	25	0	rs4461039	5.59E-06
AAD	17q21.1	2	0	rs3936197	3.98E-06
AAD	19p13.3	1	0	rs75443592	6.01E-06
AAD	19p13.11	1	0	rs138497565	1.91E-06
AAD	19q13.2	18	0	rs35493131	2.22E-06
AAD	21q22.13	1	0	rs9979967	6.07E-06

**FIGURE 2:**
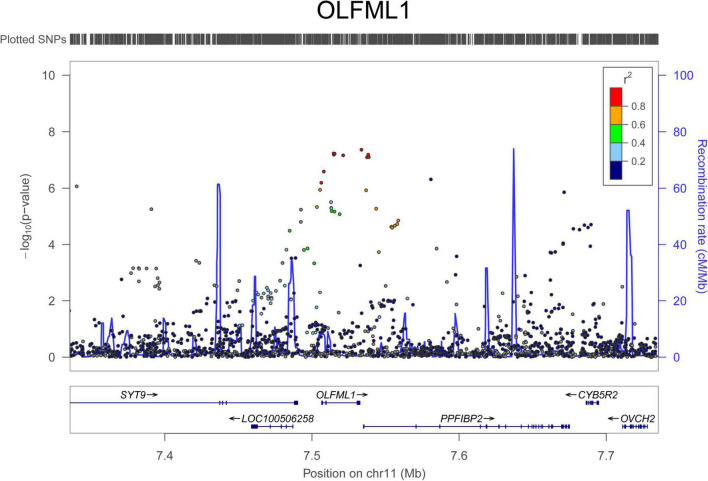
Regional association signals of *OLFML1* SNPs with AAD. The regional association plots show the associations between SNPs in *OLFML1* and AAD. The association data were downloaded at http://geneatlas.roslin.ed.ac.uk/downloads/. SNPs with minor allele frequencies greater than 0.05 in this dataset were used.

**FIGURE 3:**
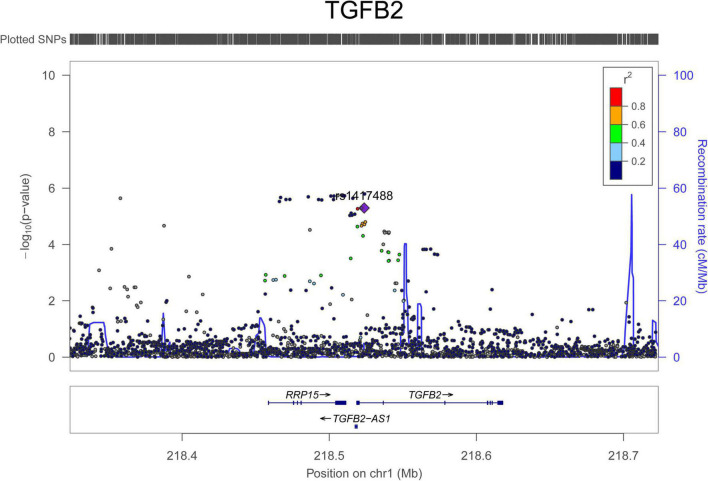
Regional association signals of *TGFB2* SNPs with AAD. The regional association plots show the associations between SNPs in *TGFB2* and AAD. The association data were downloaded at http://geneatlas.roslin.ed.ac.uk/downloads/. SNPs with minor allele frequencies greater than 0.05 in this dataset were used.

For AAD associations, 234 SNPs in 29 genomic loci that passed 1.0 × 10^–5^ were identified from the UK Biobank dataset, and 4 SNPs (rs76936122, rs76560573, rs112809417, and rs75137218) in the *OLFML1* gene region (11p15.4) passed 5.0 × 10^–8^. There were 50 SNPs nominally associated with AAD in this locus and the top SNP was rs112809417 (*P* = 4.10 × 10^–8^) (Figure 2). SNPs in the rest loci did not reach the genome-wide significance level. Nominal significant SNPs rs6658835 (5.39 × 10^–6^) and rs1417488 (5.05 × 10^–6^) were found in the *TGFB2* gene (Figure 3).

### Potential Proteins Related to Spontaneous Coronary Artery Dissection and Aneurysm and Dissection

**FIGURE 4:**
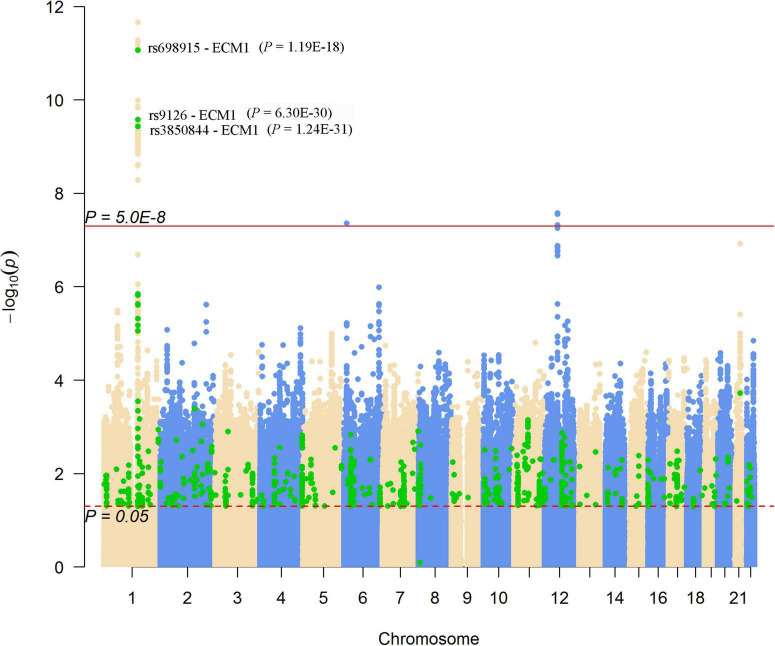
Genome-wide results for the associations between SCAD-SNPs and circulating protein levels. The Manhattan plot shows -log_10_*P*-values for the associations between genome-wide SNPs and SCAD. The data were extracted from the SCAD GWAS published in 2020 (17). The red line indicates the genome-wide significance level (*P* = 5.0 × 10^–8^). The red dotted line indicates the nominal significance level (*P* = 0.05). The green points highlight SNPs that were associated with plasma proteins with *P* < 0.05. Significant associations (*P*-values presented in parentheses) between SNPs and the plasma level of ECM1 were annotated.

**FIGURE 5:**
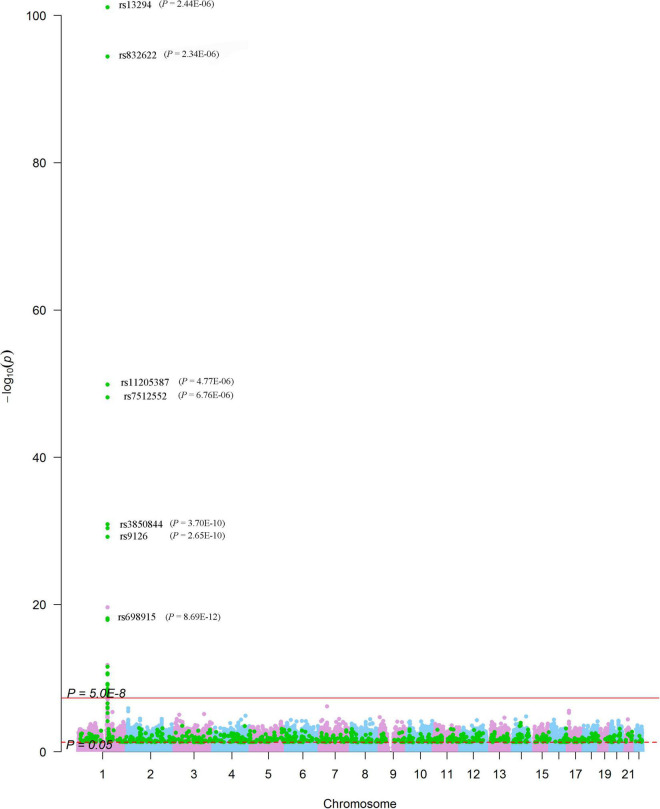
Genome-wide associations between SNPs and plasma levels of ECM1. The Manhattan plot shows -log_10_*P* values for the associations between genome-wide SNPs and plasma levels of ECM1. The data were extracted from the pQTL study conducted by Suhre et al. (24). The red line indicates the genome-wide significance level (*P* = 5.0 × 10^–8^). The red dotted line indicates the nominal significance level (*P* = 0.05). The green points highlight SNPs that were associated with SCAD with *P* < 0.05. SNPs significantly associated with SCAD were annotated (*P*-values presented in parentheses).

**FIGURE 6:**
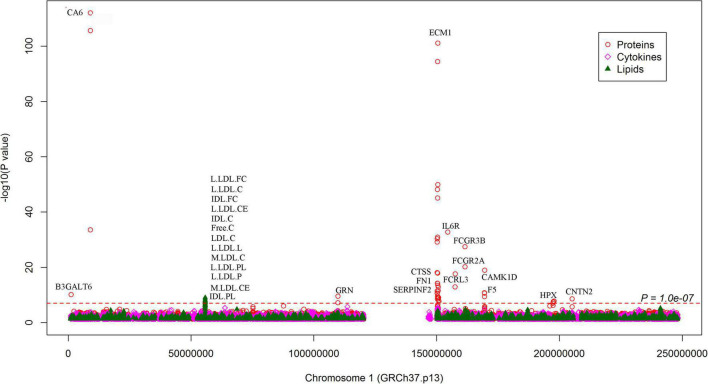
Associations between SNPs on chromosome 1 and circulating molecule levels. The Manhattan plot shows -log_10_*P*-values for the associations between genome-wide SNPs and plasma levels of proteins, cytokines, and metabolites. The data were extracted from the 7 pQTL studies. The red dotted line indicates *P* = 1.0 × 10^–7^. All SNPs plotted were associated with SCAD with *P* < 0.05. The top molecules were annotated.

We looked for pQTL signals in five studies. We found significant signals in two studies (24, 25) for SNPs in 1q21.2 (Figure 4). The SCAD-associated SNPs in 1q21.2 were strongly associated with circulating extracellular matrix protein 1 (ECM1). Among these pQTLs, eight were from the KORA study (24), including the association between rs9126, rs7512552, rs698915, rs832622, rs3850844, rs12758270, rs11205387, and rs13294 and circulating ECM1. The strongest signal was the association between rs13294 and ECM1 (*P* = 7.73 × 10^–102^), followed by rs832622 (*P* = 3.91 × 10^–95^) (Figure 5). However, the associations between these two SNPs and SCAD were not genome-wide significant (*P* = 2.44 × 10^–6^ and 2.34 × 10^–6^, respectively). Among the genome-wide significant SCAD-associated SNPs, rs698915, rs9126, and rs3850844 were significantly associated with circulating levels of ECM1 (Figure 4). In another study (25), we found significant associations between rs1694369 (SCAD association *P* = 1.45 × 10^–6^) and circulating levels of SERPINF2 (*P* = 2.69 × 10^–10^), FN1 (*P* = 6.24 × 10^–15^), and ECM1 (*P* = 1.01 × 10^–45^) (Figure 6).

In addition to the genome-wide significant SNPs, a total of 184,983 SNPs, which were nominally (*P* < 0.05) associated with SCAD, were associated with 637 proteins (Figure 4). On chromosome 1, many nominal SCAD-associated SNPs were strongly associated with circulating protein levels (Figure 6). For example, rs3765963 (an intronic SNP in the *CA6* gene) at 1p36.23 was strongly associated with the circulating level of CA6 (*P* = 8.70 × 10^–113^); rs4584384 (intronic SNP in the *TDRD10* gene) at 1q21.3 was strongly associated with circulating levels of IL6R (*P* = 1.84 × 10^–33^).

**FIGURE 7:**
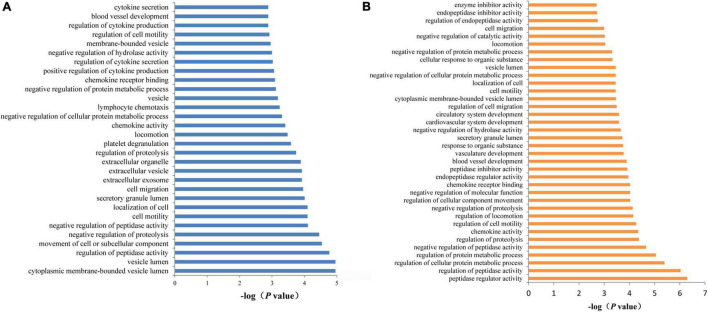
Functional annotation enrichment analysis of SCAD-related proteins. **(A)** There were 30 statistically significant GO terms that were enriched in AD-related proteins. **(B)** The 26 proteins (ADAMTS4, DDC, B2M, CCL20, CRISP3, ECM1, and TSHB) that may be affected by SNPs at 1q21.2 were enriched in 36 GO terms.

To examine the potential biological functions of the proteins affected by the SCAD-associated SNPs, we performed functional enrichment analysis in the DAVID database. To incorporate as many proteins as possible, we set a less stringent threshold of 0.01 for pQTLs. Subsequently, a total of 54 proteins were selected. These 54 associated proteins were enriched in 30 GO terms (false discovery rate < 0.05), including cellular components such as vesicle lumen (*P* = 1.10 × 10^–5^), secretory granule lumen (*P* = 9.80 × 10^–5^), and extracellular exosome (*P* = 1.20 × 10^–4^); biological processes such as regulation of peptidase activity (*P* = 1.70 × 10^–5^) and movement of cell or subcellular component (*P* = 2.90 × 10^–5^); and molecular functions such as chemokine activity (*P* = 3.90 × 10^–4^) and chemokine receptor binding (*P* = 8.10 × 10^–4^) (Figure 7A). Concentrations of 26 proteins (encoded by *CTSS*, *ECM1*, *CAT*, *CNDP1*, *KNG1*, *SLAMF7*, *TIE1*, *CXCL1*, *MBL2*, *ESD*, *CXCL16*, *CCL14*, *KCNE5, CST7*, *PSME1*, *GPC3*, *MAP2K4*, *SPOCK3*, *LRPPRC*, *CLEC4M*, *NOG*, *C1QTNF9*, *CX3CL1*, *SCP2D1*, *SERPINF2*, and *FN1*) were affected by genetic variants at 1q21.2. These proteins were enriched in biological processes such as regulation of peptidase activity (*P* = 9.40 × 10^–7^), regulation of cellular protein metabolic process (*P* = 4.10 ×10^–6^), regulation of protein metabolic process (*P* = 2.20 × 10^–5^); molecular function such as peptidase regulator activity (*P* = 5.10 × 10^–7^), and chemokine activity (*P* = 4.50 × 10^–5^); cellular component such as secretory granule lumen (*P* = 1.90 × 10^–4^) and cytoplasmic membrane-bounded vesicle lumen (*P* = 3.40 × 10^–4^), and so on (Figure 7B).

A total of 64 proteins were found to be associated with AAD-associated SNPs. Proteins (FGF6, FGF9, HGF, BCL2L1, and VEGFA) involved in the Ras signaling pathway were identified. The genome-wide significant SNPs in *OLFML1* were associated with circulating levels of SLURP1, PLAU, CDKN1B, ACVR1B, and STAB2. Furthermore, we performed MR analysis to evaluate the associations between the identified proteins and AAD. Circulating ECM1 levels were nominally associated with AAD in IVW (*P* = 0.0129) and MR-PRESSO (*P* = 0.0193) analyses. Circulating SPOCK3 levels were associated with AAD in weighted median (*P* = 0.0024), IVW (*P* = 0.0013), and MR-PRESSO (*P* = 6.94 × 10^–4^) analyses.

### Potential Cytokines Related to Spontaneous Coronary Artery Dissection and Aneurysm and Dissection

For SNPs nominally associated with SCAD (*P* < 0.05), we found 220,594 SNPs that were nominally associated with circulating levels of 42 cytokines. For SNPs associated with SCAD at *P* < 1.0 × 10^–5^, we found that rs1631973 (SCAD association *P* = 1.49 × 10^–6^) and rs9436119 (SCAD association *P* = 1.52 × 10^–6^) were associated with cytokine bNGF levels (*P* = 7.44 × 10^–6^ and 3.91 × 10^–6^, respectively). In addition, rs7764227 (SCAD association *P* = 5.34 × 10^–3^) and rs9369433 (SCAD association *P* = 5.82 × 10^–3^) were associated with circulating levels of IL10 (*P* = 7.16 × 10^–32^ and 7.96 × 10^–32^, respectively), IL12p70 (*P* = 1.78 × 10^–48^ and 9.62 × 10^–49^, respectively), IL13 (*P* = 1.34 × 10^–28^ and 6.04 × 10^–29^, respectively), IL7 (*P* = 3.35 × 10^–19^ and 2.53 × 10^–19^, respectively), and VEGF (*P* = 1.17 × 10^–72^ and 6.66 × 10^–73^, respectively).

For SNPs associated with AAD at *P* < 1.0 × 10^–5^, we found few significant signals for cytokines. rs10514926 (AAD association *P* = 1.90 × 10^–7^) and rs141292109 (AAD association *P* = 1.56 × 10^–7^) were associated with IL7 (*P* = 3.92 × 10^–5^ and 3.94 × 10^–5^, respectively). A total of 28 SNPs were nominally associated with the IL1b level (*P* < 0.05). In the MR analysis, the IL1b level was nominally associated with AAD in the weighted median (*P* = 0.0031), IVW (*P* = 0.0092), MR–Egger (*P* = 0.0422), and MR-PRESSO (*P* = 0.0165) analyses.

### Lipid Metabolites Related to Spontaneous Coronary Artery Dissection and Aneurysm and Dissection

**FIGURE 8:**
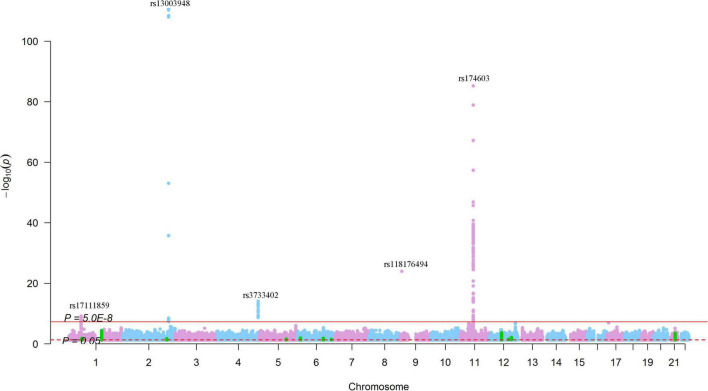
Genome-wide results for the associations between SCAD-SNPs and circulating levels of metabolites. The Manhattan plot shows -log_10_*P*-values for the associations between genome-wide SNPs and circulating levels of metabolites. The data were extracted from a systematic metabolomics study (34). The red line indicates the genome-wide significance level (*P* = 5.0 × 10^–8^). The red dotted line indicates the nominal significance level (*P* = 0.05). The green points highlight SNPs that were associated with SCAD with *P* < 1.0 × 10^–5^. The top SNPs were annotated.

For SNPs nominally associated with SCAD (*P* < 0.05), we found 48,689 SNPs that were nominally associated with circulating levels of the 123 circulating metabolic traits (Figure 8). The top signals were found for the associations between rs13003948 (SCAD association *P* = 8.92 × 10^–3^), rs6717111 (SCAD association *P* = 6.08 × 10^–3^), rs4310999 (SCAD association *P* = 8.63 × 10^–3^), rs13029304 (SCAD association *P* = 6.86 × 10^–3^), rs4630697 (SCAD association *P* = 6.98 × 10^–3^),and Gly (*P* = 1.24 × 10^–111^, 9.36 × 10^–109^, 5.12 × 10^–111^, 4.88 × 10^–111^, and 3.54 × 10^–109^, respectively), followed by the associations between rs174603 (SCAD association *P* = 7.66 × 10^–3^) and rs11230815 (SCAD association *P* = 1.03 × 10^–3^) and otPUFAs (*P* = 6.12 × 10^–86^ and 1.38 × 10^–47^, respectively). For SNPs associated with SCAD with *P* < 1.0 × 10^–5^, no SNP was associated with metabolites at *P* < 1.0 × 10^–5^, but there were 329 SNPs associated with 85 metabolites at *P* < 0.05 (Figure 8). These results suggested that metabolite associations clustered at 1q21.2, 12q13.3, and 21q22.11. SNPs at 1q21.2 were associated with 13 metabolites (*P* < 1.0 × 10^–7^) (Figure 6). The SCAD-associated SNP rs7366347 (SCAD association *P* = 1.44 × 10^–9^) was strongly associated with circulating levels of LDL-D (*P* = 3.99 × 10^–5^). In addition to rs7366347, 281 SNPs (SCAD association *P* < 1.0 × 10^–5^) at 1q21.2 were associated with circulating levels of LDL-D (*P* < 0.05).

Associations between AAD-associated SNPs and 122 metabolic traits were found. The top signal was found for otPUFA. For SNPs associated with AAD at *P* < 5.0 × 10^–5^, no SNP was associated with metabolites at *P* < 1.0 × 10^–5^. However, for SNPs nominally associated with AAD, more than 500 association signals were found for metabolites such as L.HDL.P, L.HDL.L, HDL.C, L.HDL.PL, L.HDL.CE, M.VLDL.P, XL.HDL.TG, XS.VLDL.L, APOB, XS.VLDL.P, S.VLDL.FC, L.HDL.C, S.VLDL.C, L.HDL.FC, L.VLDL.CE, IDL.TG, M.VLDL.TG, and XS.VLDL.TG.

**FIGURE 9:**
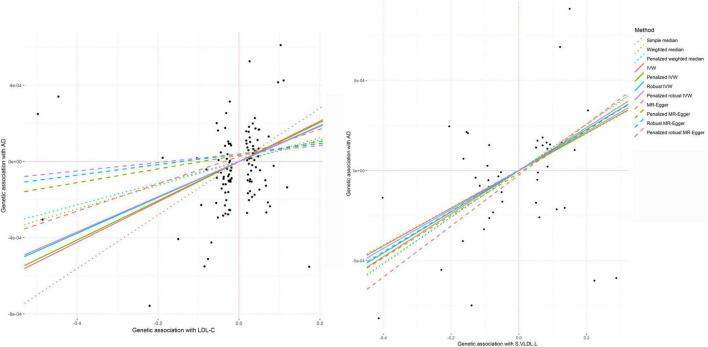
The causal associations between LDL-c and S.VLDL.L levels and AAD.

In the MR analysis, LDL-c levels were associated with AAD in the weighted median (*P* = 7.80 × 10^–3^), IVW (*P* = 1.17 × 10^–4^), and MR-PRESSO (*P* = 1.94 × 10^–4^) analyses (Figure 9). TC levels were associated with AAD in IVW (*P* = 1.21 × 10^–3^), MR–Egger (*P* = 2.63 × 10^–3^), and MR-PRESSO (*P* = 3.97 × 10^–4^) analyses. HDL-c and TG were found to be associated with AAD in the weighted median (*P* = 7.50 × 10^–3^ and 2.11 × 10^–2^, respectively), IVW (*P* = 7.49 × 10^–4^ and 3.88 × 10^–3^, respectively), and MR-PRESSO (*P* = 9.89 × 10^–4^ and 4.77 × 10^–3^, respectively) analyses. We also found that high cholesterol and lipidemia were significantly associated with AAD in weighted median (*P* = 7.84 × 10^–6^ and 2.30 × 10^–9^, respectively), IVW (*P* = 6.26 × 10^–13^ and 8.83 × 10^–10^, respectively), and MR-PRESSO (*P* = 1.34 × 10^–14^ and 1.30 × 10^–15^, respectively) analyses, and nominal associations in MR–Egger (*P* = 4.24 × 10^–4^ and 8.46 × 10^–3^, respectively) analyses were found. We performed a multivariable MR analysis for the association between circulating levels of LDL-c, HDL-c, TC and TG and AAD. After adjusting for HDL-c or TG, LDL-c was associated with AAD (*P* = 0.002 and 0.039, respectively). When adjusted for TC, the association between LDL-c and AAD was not significant.

Circulating levels of otPUFA were associated with AAD in weighted median (*P* = 2.91 × 10^–3^), IVW (*P* = 8.92 × 10^–4^) and MR-PRESSO (*P* = 1.34 × 10^–3^) analyses. In addition, total lipids in small very-low-density lipoprotein particles (S.VLDL.L) level was significantly associated with AAD (*P* IVW = 2.93 × 10^–5^, *P* MR–Egger = 5.11 × 10^–3^) (Figure 9). Other very-low-density lipoprotein particles such as S.VLDL.C (*P* IVW = 9.54 × 10^–4^, *P* MR–Egger = 8.56 × 10^–3^), S.VLDL.FC (*P* IVW = 1.10 × 10^–4^, *P* MR–Egger = 8.22 × 10^–3^), S.VLDL.PL (*P* IVW = 1.20 × 10^–3^, *P* MR–Egger = 9.06 × 10^–4^), and XS.VLDL.TG (*P* IVW = 1.99 × 10^–4^, *P* MR–Egger = 3.14 × 10^–2^) were also found to be associated with AAD. The association between S.VLDL.L (*P* = 9.05 × 10^–5^), S.VLDL.C (*P* = 1.48 × 10^–3^), S.VLDL.FC (*P* = 2.49 × 10^–4^), S.VLDL.PL (*P* = 1.96 × 10^–3^), XS.VLDL.TG (*P* = 4.01 × 10^–4^) and AAD were significant in MR-PRESSO analyses. In multivariable MR analysis, the association between circulating levels of S.VLDL.L and AAD was not significant after adjusting for S.VLDL.FC or XS.VLDL.TG.

### Potential Causal Variants

We searched for several kinds of functional variants among pQTLs that may affect circulating proteins and metabolites in public databases. We found that rs12758270 (3′-UTR) in *RPRD2* and rs3818978 (5′-UTR) in *MRPS21* were associated with m^6^A methylation. The SNP rs12758270 was significantly associated with *ECM1* mRNA expression levels in fibroblast cells (*P* = 2.79 × 10^–5^) and rs3818978 was significantly associated with *ADAMTSL4* and *MRPS21* gene expression levels in aortic artery tissue (*P* = 8.31 × 10^–7^ and 2.30 × 10^–7^, respectively). The SNP rs580060 (3′-UTR) in *MRPS21* was associated with A-to-I RNA methylation and *ADAMTSL4* mRNA levels in aortic artery tissue (*P* = 1.27 × 10^–6^).

The missense variants rs3737240 [T (ACG) > M (ATG)] in the sixth exon and rs13294 [G (GGT) > S (AGT)] in the eighth exon of *ECM1* were associated with *ADAMTSL4* mRNA levels in aortic artery tissue (*P* = 1.28 × 10^–5^ and 6.70 × 10^–6^, respectively) and may have impacts on the phosphorylation of ECM1 protein.

The *ALK* gene variant rs4535004 (*P* = 2.60 × 10^–4^) and the *RPRD2* gene variants rs7002 (3′-UTR, *P* = 5.60 × 10^–5^) and rs834230 (*P* = 2.62 × 10^–4^) altered the activity of putative regulatory elements in K562 cells. The *RPRD2* gene variant rs11205370 (*P* = 8.08 × 10^–4^) altered the activity of putative regulatory elements in HepG2 cells. Among these four regulatory SNPs, rs7002 (*P* = 1.16 × 10^–6^), rs834230 (*P* = 1.83 × 10^–6^), and rs11205370 (*P* = 8.23 × 10^–7^) were associated with *ADAMTSL4* mRNA levels, and rs11205370 (*P* = 5.35 × 10^–7^) was associated with *MRPS21* gene expression levels in aortic artery tissue.

## Discussion

This study searched for potential molecules that were related to SCAD and AAD by integrating data from GWAS and pQTL studies. We found proteins, cytokines and metabolites for SCAD and AAD and highlighted pQTLs at 1q21.2 that were strongly associated with circulating levels of ECM1. Moreover, this study also found potential causal proteins and lipid metabolites for AAD.

In our study, we identified proteins that may be regulated by genetic variants associated with both proteins and SCAD and AAD based on pleiotropic associations. A notable signal for ECM1 was found. The *ECM1* gene is located at chromosome 1q21. This gene encodes the ECM1 protein, a widely expressed 85-kDa glycoprotein (48). ECM1 protein in the heart is derived from infiltrating inflammatory cells and assists cardiac fibrosis through ERK1/2 and/or Akt activation (49). Inflammatory cells play roles in ECM remodeling and fibrosis in wound healing in ischemic infarcts (50–52). ECM1 interacts with the majority of other ECM proteins to enhance ECM protein binding and mediate various biological processes (53), such as regulation of T helper lymphocyte migration (54) and endothelial cell proliferation (55) and inhibition of MMP9 proteolytic activity (56). ECM1 is associated with cardiac aging and MI (49). As we showed in the present study, genetic variants at 1q21.2, which have been confirmed to be associated with SCAD, were strongly associated with circulating levels of ECM1. Furthermore, circulating levels of ECM1 were found to be associated with AAD in MR analysis. Therefore, circulating ECM1 may play a role in the development of SCAD and AAD.

The present study suggested that LDL-c and other very-low-density lipoprotein particles were causally associated with AAD. Elevated plasma levels of LDL-c result in atherogenesis, which promotes AAD development (29). The early stage of atherosclerosis development begins with vascular endothelial dysfunction and intima-media thickness (57). The complex process may result from the presence of highly toxic oxidized LDL particles and a progressive accumulation of cholesterol and other lipids in the intimal-medial layer of the aorta with secondary inflammation, repetitive fibrous tissue deposition, and eventually luminal surface erosions and the appearance of often mobile thrombi protruding into the lumen of the aorta (58). Lymphocytes and monocytes penetrate the damaged endothelial lining, where they become macrophages laden with LDL-c and then become foam cells (59). Atherosclerosis may lead to aortic wall fragility, including calcification, fibrosis, extracellular matrix degradation, intimal thickening and extracellular fatty acid deposition, which compromise the elastic properties of the aortic wall (60). Atherosclerosis primarily affects the coronary arteries but may also contribute to the development of AAD (61). The atheroma is composed of a core of fatty substances separated from the lumen by collagen and smooth muscle. Chronic inflammation within the atheroma results in plaque instability and subsequent rupture (62, 63). Dyslipidemia is one of the most important modifiable lifestyle risk factors for AAD. Eradication of dyslipidemia is presumably equally important to smoking cessation in AAD prevention (60). The findings of this study indicated that lipids play a causal role in AAD and suggested the importance of blood lipid regulation in SCAD and AAD prevention and treatment.

We identified many putative functional SNPs among the pQTLs. Two SNPs, rs12758270 (3’-UTR) in *RPRD2* and rs3818978 (5′-UTR) in *MRPS21*, have the potential to affect m^6^A methylation in the mRNA of *RPRD2* and *MRPS21*. The associations between rs12758270 and *ECM1* mRNA expression levels in fibroblast cells and between rs3818978 and *ADAMTSL4* and *MRPS21* gene expression levels in aortic artery tissue support their potential functions. The SNP rs580060 in the 3′-UTR of *MRPS21* may affect A-to-I RNA methylation and was associated with *ADAMTSL4* mRNA levels in aortic artery tissue. In addition, missense variants rs3737240 and rs13294 in *ECM1* were associated with *ADAMTSL4* mRNA levels in aortic artery tissue. Moreover, rs3737240 and rs13294 are phosphorylation-associated SNPs that may have impacts on the phosphorylation of ECM1 protein, and rs13294 was found to be associated with circulating ECM1 levels. These functional SNPs may alter protein levels and subsequently affect SCAD and AAD risk. However, how these SNPs participate in the pathogenesis of SCAD and AAD by altering circulating proteins needs to be elucidated in future studies.

## Conclusion

This study showed that SCAD-associated and AAD-associated SNPs may have strong effects on circulating levels of proteins and metabolites. Therefore, this study found circulating proteins and metabolites that were pleiotropically associated with AAD. Circulating ECM1 and LDL-c may play important roles in the pathology of AAD. Genetic variants at 1q21.2 may have regulatory potential to affect gene expression in related tissues, such as aortic artery and circulating protein levels, and subsequently affect SCAD and AAD risk. This study increased our understanding of the regulatory roles of genetic variants in SCAD and AAD susceptibility genes. The findings highlighted potential pathways for further detection of functional mechanisms underlying the associations between genetic variants and SCAD and AAD.

## Data Availability Statement

The original contributions presented in the study are included in the article/supplementary material, further inquiries can be directed to the corresponding author/s.

## Ethics Statement

The studies involving human participants were reviewed and approved by the Fujian Medical University. The patients/participants provided their written informed consent to participate in this study.

## Author Contributions

TC and LC designed this study. TC, XY, and ZQ collected the data. TC was responsible for the statistical analysis. MT and TC wrote the draft. MT, XL, and LC revised this draft. LC finalized this manuscript. All authors read and approved the final manuscript.

## Conflict of Interest

The authors declare that the research was conducted in the absence of any commercial or financial relationships that could be construed as a potential conflict of interest.

## Publisher’s Note

All claims expressed in this article are solely those of the authors and do not necessarily represent those of their affiliated organizations, or those of the publisher, the editors and the reviewers. Any product that may be evaluated in this article, or claim that may be made by its manufacturer, is not guaranteed or endorsed by the publisher.

## Footnotes

1
http://geneatlas.roslin.ed.ac.uk/downloads/

2
http://www.phpc.cam.ac.uk/ceu/proteins/

3
https://zenodo.org/record/264128#.X3u5ga95uUk

4
https://www.decode.com/summarydata/

5
https://david.ncifcrf.gov/

6
http://rmvar.renlab.org/

7
http://phossnp.biocuckoo.org/index.php

8
https://sure.nki.nl/
